# Lipoprotein (a) upregulates ABCA1 in liver cells via scavenger receptor-B1 through its oxidized phospholipids[S]

**DOI:** 10.1194/jlr.M056150

**Published:** 2015-07

**Authors:** Monika Sharma, Anne Von Zychlinski-Kleffmann, Carolyn M. Porteous, Gregory T. Jones, Michael J. A. Williams, Sally P. A. McCormick

**Affiliations:** † Departments of Surgical Sciences University of Otago, Dunedin, New Zealand; § Medicine, Dunedin School of Medicine, University of Otago, Dunedin, New Zealand; * Department of Biochemistry, University of Otago, Dunedin, New Zealand

**Keywords:** adenosine 5′-triphosphate binding cassette transporter A1, cholesterol efflux, high density lipoprotein, nuclear receptor/liver X receptor, nuclear receptor/peroxisome proliferator-activated receptor

## Abstract

Elevated levels of lipoprotein (a) [Lp(a)] are a well-established risk factor for developing CVD. While Lp(a) levels are thought to be independent of other plasma lipoproteins, some trials have reported a positive association between Lp(a) and HDL. Whether Lp(a) has a direct effect on HDL is not known. Here we investigated to determine whether Lp(a) had any effect on the ABCA1 pathway of HDL production in liver cells. Incubation of HepG2 cells with Lp(a) upregulated the PPARγ protein by 1.7-fold and the liver X receptor α protein by 3-fold. This was accompanied by a 1.8-fold increase in ABCA1 protein and a 1.5-fold increase in cholesterol efflux onto apoA1. We showed that Lp(a) was internalized by HepG2 cells, however, the ABCA1 response to Lp(a) was mediated by the selective uptake of oxidized phospholipids (oxPLs) from Lp(a) via the scavenger receptor-B1 and not by Lp(a) internalization per se. We conclude that there is a biological connection between Lp(a) and HDL through the ability of Lp(a)’s oxPLs to upregulate HDL biosynthesis.

Lipoprotein (a) [Lp(a)] consists of a LDL linked by a disulphide bond to apo(a) (1). Elevated levels of Lp(a) (>50 mg/dl) are an independent risk factor for developing CVD (2). Lp(a) levels show a strong correlation with oxidized phospholipids (oxPLs) in plasma, more so than LDL (3). Indeed the apo(a) moiety has been shown to bind oxPLs (4). Oxidized lipids activate a number of key molecules involved in atherosclerosis, including the PPARγ and liver X receptor (LXR)α transcription factors (5). PPARγ and LXRα upregulate scavenger receptors on macro­phages to promote foam cell formation in the artery but also upregulate the ABCA1 protein to promote cholesterol efflux (5). In liver cells, PPARγ and LXRα promote ABCA1-mediated cholesterol efflux to stimulate HDL formation (6–8).

Most, but not all, clinical studies show Lp(a) levels to be independent from those of other plasma lipoproteins (2, 9). Detecting associations with Lp(a) is difficult because plasma levels vary hugely within any given population (over 1,000-fold) and are skewed in distribution toward low levels (2, 10, 11). A positive association between HDL and Lp(a) levels has been reported in different epidemiological studies (12–14), but whether Lp(a) has any direct biological effect on HDL is unknown. A recent study has documented that introduction of low levels of Lp(a) into mice results in an increase in the HDL fraction (15). Past studies have shown that LDL induces ABCA1-mediated cholesterol efflux in both macrophage (16) and endothelial cells (17). As Lp(a) is structurally similar to LDL, one might predict that it may also promote ABCA1-mediated intracellular cholesterol efflux and could therefore promote HDL production.

The liver is the major site for Lp(a) catabolism (18) and HDL production (8). The cell surface receptors responsible for Lp(a) uptake in the liver are not completely understood. Members of the LDL receptor (LDLR) family have been implicated in Lp(a) uptake. The LDLR has been associated with Lp(a) uptake in familial hypercholesterolemia patients (19), but definite proof of Lp(a) uptake by the LDLR in the liver is lacking. The VLDL receptor (VLDLR) is known to internalize Lp(a) (20), but is not expressed by the liver. Kostner and colleagues reported that a significant amount of Lp(a) uptake in the liver is mediated by the asialoglycoprotein receptor (21); however, subsequent in vivo experiments have questioned this (18). A recent publication demonstrated enhanced uptake of Lp(a) in cell lines and in transgenic mice over­expressing scavenger receptor-B1 (SR-B1) in the liver, providing evidence that SR-B1 is a receptor for Lp(a) (22).

Here we investigated whether Lp(a) had any effect on the ABCA1-mediated pathway of cholesterol efflux in liver cells in order to establish whether there was a biological connection between Lp(a) and HDL. Our results showed that Lp(a) stimulates ABCA1 via activation of PPARγ and LXRα resulting in a functional effect of enhanced cholesterol efflux to apoA1. This prompted us to further investigate the role of SR-B1 and Lp(a) holoparticle uptake in promoting the ABCA1 response.

## MATERIALS AND METHODS

### Lp(a) isolation

LDL and Lp(a) were isolated from a pooled plasma sample made from equal amounts of plasma obtained from five healthy individuals with Lp(a) levels >50 mg/dl after informed consent. Ethical approval for the study was granted by the Lower Regional South Ethics committee. The lipid levels and apo(a) isoform sizes of the five samples are given in **Table 1**. A pooled plasma sample was used to avoid any bias from using Lp(a) containing just one apo(a) isoform. LDL and Lp(a) were isolated from the pooled plasma using a combination of density ultracentrifugation (23) and fast protein LC (FPLC) (24). Briefly, 4 ml of plasma was adjusted to a density of 1.063 g/ml with KBr and the adjusted plasma was overlaid with 1.063 g/ml KBr solution and subjected to density ultracentrifugation in a Beckman NVTi65 rotor for 3 h at 10°C at 60,000 rpm. The top layer containing VLDL and LDL was fractionated by FPLC to isolate LDL from VLDL. The remaining plasma was adjusted to a density of 1.12 g/ml with KBr and subjected to density ultracentrifugation at 100,000 rpm for 4 h at 10°C using a Beckman TL-100.3 ultracentrifuge. The top layer containing the 1.063–1.12 g/ml density fraction was fractionated by FPLC to isolate Lp(a) from HDL and residual plasma proteins. Isolation of LDL and Lp(a) was also performed using 25 μM butylated hydroxytoluene (BHT) and 0.26 mM EDTA throughout the process to establish the effect on oxidized lipid content on both lipoproteins as measured by the thiobarbituric acid reactive substance (TBARS) assay. The purity of the isolated LDL and Lp(a) was checked by lipoprotein electrophoresis on LIPO + Lp(a) hydragels using the HYDRASYS agarose gel electrophoresis system (Sebia, Norcross, GA). Gels were stained with Fat Red 7B and subjected to Western blotting with anti-apo(a), anti-apoB, and anti-apoA1 antibodies. Lp(a) and LDL were quantified by measuring the total protein content of each using the Qubit^®^ protein assay kit (Life Technologies, Carlsbad, CA).

**TABLE 1.** Lipid levels and apo(a) isoform sizes of plasma samples

Sample Identification	Lp(a) (mg/dl)	Apo(a) Isoform Size	HDL (mM)	LDL (mM)
151	109	19	1.91	1.7
172	158	20, 19	1.36	2.3
228	109	22, 18	1.44	5.3
229	122	17	1.72	2.2
230	89	19	1.36	2.7

Lp(a) levels were measured by immunoassay [QUANTIA Lp(a), Abbott Diagnostics, Abbott Park, IL]. Lipid levels were determined by enzymatic assay (Roche). The apo(a) isoform size was determined by electrophoresis on 4% polyacrylamide gels followed by immunoblotting (42) and is given as the number of KIV_2_ repeats relative to apo(a) isoform standards.

### Cell culture

Hepatocellular carcinoma (HepG2) cells and human hepatoma (Hep3B) cells were obtained from American Type Culture Collection (Manassas, VA). Cells were maintained in advanced DMEM supplemented with 10% fetal bovine serum (Bio International, Auckland, New Zealand), 2 mM L-glutamine, 0.25 μg/ml amphotericin B, 100 U/ml penicillin, and 100 μg/ml streptomycin (Invitrogen, Carlsbad, CA) at 37°C in a humidified environment with 5% CO_2_. HepG2 and Hep3B cells were checked for apo(a) expression by Western blot on cell lysate and media which showed the absence of apo(a) in both. Twenty-four hours after seeding HepG2 cells at 5 × 10^5^ density, cells were treated with either purified Lp(a) or LDL at 1, 5, and 10 μg/ml for 12 h in DMEM containing 1% serum. Hep3B cells were seeded at 2.5 × 10^5^ density and treated with purified Lp(a) at 5 μg/ml for 12 h. The trypan blue dye exclusion test (25) was performed to check the viability of cells after Lp(a) treatment and we found the viability to be 70–80% with no significant effect of Lp(a) concentration.

### RT-PCR

Total RNA was extracted from Lp(a)-treated HepG2 cells using the RNeasy Mini kit (Qiagen, Venlo, Limburg) as per the manufacturer’s instructions. On-column DNase digestion was performed using the RNase-free DNase set (Qiagen). cDNA was synthesized from 1 μg RNA using the First Strand cDNA synthesis kit from Roche (Basel, Switzerland). Quantitative RT-PCR was performed using the KAPA SYBR^®^ FAST Universal 2× qPCR Master Mix (Kapa Biosystems, Wilmington, MA) and cDNA as the template on a LightCycler^®^ 480 (Roche). Specific primers spanning exonic regions in target genes, ABCA1, LXRα, PPARγ, and reference genes, β-2 microglobulin and GAPDH were designed (see supplementary Table 1 for primer sequences). Expression of each target gene was quantified using C_t_ values after normalizing to the two reference genes.

### Western blot

HepG2 cells were harvested in RIPA buffer [50 mM Tris-HCl (pH 7.8), 150 mM NaCl, 0.1% SDS, 0.5% sodium deoxycholate, 1% Triton X-100] supplemented with complete mini protease inhibitors (Roche). Cell lysates containing 40 μg protein were resolved on 4% polyacrylamide gels for apo(a), 7.5% gels for ABCA1, or on 10% gels for LXRα, PPARγ, and actin. Proteins were transferred to nitrocellulose membrane. After blocking with 5% skim milk, membranes were incubated with primary antibodies specific for ABCA1 (ab7630), LXRα (ab28478), PPARγ (ab27649) (all from Abcam, Cambridge, England), actin (A5060, Sigma, St. Louis, MO), and the apo(a)-specific antibody, a5-hp (26), at 4°C overnight. After incubation with the appropriate secondary antibody, membranes were developed with ECL reagents and imaged with a Fujifilm LAS3000 (R&D Systems, Minneapolis, MN). The quantification of blots was done using the ImageQuant TL software (Amersham Biosciences, Piscataway, NJ) with proteins normalized against actin.

### TBARS assay

The concentration of lipid oxidation products in the LDL and Lp(a) preparations was measured using the (TBARS) (27) assay, which detects malondialdehyde (MDA) generated from oxidized lipids. The TBA-MDA adduct was measured by fluorometric analysis (excitation, 544 nm; emission, 590 nm) in LDL and Lp(a) samples using a POLARstar Optima (BMG Labtech, Offenburg, Germany).

### Promoter activity assay

ABCA1 promoter activity was checked by luciferase assay (28). HepG2 cells were transfected with 1 μg of the pGL4.10 luciferase reporter vector containing 700 bp of the ABCA1 promoter region (from −1 to −699 bp) and 10 ng of internal control Renilla vector (phRL-SV40) for 48 h using FuGENE^®^ HD transfection reagent (Roche). Forty-eight hours after transfection, HepG2 cells were treated with 1, 5, and 10 μg/ml Lp(a) for 12 h in serum-free DMEM. Cell lysates were assayed for Renilla and firefly luciferase activity using the Dual Luciferase^®^ reporter assay system (Promega, Madison, WI) as per the manufacturer’s instructions. Firefly luciferase measurements were normalized to Renilla luciferase measurements.

### Cholesterol efflux assay

Cholesterol efflux assays on HepG2 cells were performed as described previously (29). Cells were seeded in 12-well plates at 2 × 10^5^ cells per well. After 24 h, cells were labeled with 0.5 μCi/ml of [1,2 ^3^H (N)]cholesterol in DMEM for another 48 h. After incubation, cells were treated with Lp(a) at 1, 5, and 10 μg/ml concentrations for 12 h in serum-free DMEM followed by treatment with 20 μg/ml purified apoA1 for 2 h. Cells in the plate were lysed with 0.1 M NaOH and Optiphase Hisafe II scintillation fluid (Perkin Elmer) was added to the medium and cell lysates. Tritium decay over 5 min in the medium and cell lysates was measured as disintegrations per minute using a liquid scintillator analyzer (Perkin Elmer, Boston, MA). Nonspecific efflux, in the absence of apoA1, was determined and subtracted from each experimental measurement. Cholesterol efflux was calculated using the following equation: cholesterol efflux = dpm(media with apoA1) − dpm(media without apoA1)/dpm(cells + media) × 100.

### Immunohistochemistry

Lp(a)-treated HepG2 cells were fixed with 4% paraformaldehyde and subsequently incubated with AlexaFluor 594 wheat germ agglutinin (WGA) (Invitrogen) membrane stain (5 μg/ml) for 10 min at room temperature. Cells were permeabilized and blocked with 3% goat serum in 0.1% Triton X-100 for 30 min at room temperature followed by an overnight incubation with an apo(a)-specific monoclonal antibody, a5 (26), at 4°C. Cells were incubated with an anti-mouse IgG AlexaFluor 488 (Invitrogen) for 2 h at room temperature. Coverslips were mounted with ProLong^®^ Gold Antifade reagent with DAPI (Invitrogen). Images were obtained using an Olympus FluoView™ FV1000 confocal microscope with the argon (488 nm) and HeNe (633 nm) lasers. A series of images along the z axis at 0.5 μm step size from top to bottom of the cell were collected. Image analysis and statistics were done using the Olympus FluoView™ FV1000 Image Examiner software.

### Antibody blocking

The SR-B1 receptor was blocked with 5 μg/ml of anti-SR-B1 antibody (Novus Biological, Littleton, CO) preincubated with HepG2 cells for 3 h at 37°C prior to Lp(a) treatment. The SR-B1 receptor was also blocked with an inhibitor, Block Lipid Transport-1, BLT-1 (Merck Millipore, Darmstadt, Germany), preincubated with HepG2 cells at 100 μM for 1 h at 37°C prior to Lp(a) treatment. Apo(a) internalization was blocked with an anti-apo(a) polyclonal antibody (Wako, Richmond, Virginia) preincubated with 5 μg/ml Lp(a) for 1 h at room temperature prior to incubation with HepG2 cells. In addition to blocking with anti-apo(a), the oxPLs on Lp(a) were blocked with 5 μg/ml of the E06 antibody (Avanti Polar Lipids, Alabaster, AL) prior to Lp(a) treatment. RNA and protein were extracted from treated cells and analyzed for the ABCA1, PPARγ, and LXRα mRNA and protein levels, as described above.

### Fluorescent lipid and protein labeling for uptake assay

Surface lipids in Lp(a) were labeled with Fast Dil-C18 (Invitrogen) by incubating 0.3 mg of Dil with 100 μg of Lp(a) overnight at 37°C and removing unincorporated Dil on a Sephadex-G-25 column. HepG2 cells were treated with 5 μg/ml of Dil-Lp(a) for 12 h with and without anti-SR-B1 and anti-apo(a) blocking. After 12 h, cells were lysed in lysis buffer (0.1% SDS and 0.1 M NaOH) and the lysate quantified for Dil fluorescence. Protein labeling of Lp(a) was done using an apo(a)-specific primary antibody, a5 (26), in combination with an AlexaFluor 488 IgG secondary antibody. HepG2 cells were treated with 5 μg/ml of Lp(a) for 12 h with and without anti-SR-B1 and anti-apo(a) blocking. After 12 h, cells were fixed, permeabilized, and probed against anti-apo(a) followed by the AlexaFluor 488 secondary antibody and fluorescence quantified using the POLARstar Optima (BMG Labtech, Offenburg, Germany).

### Statistical analysis

Data are expressed as mean ± SEM. Differences between means were analyzed by the unpaired Student’s *t*-test.

## RESULTS

### Characterization of purified Lp(a)

Agarose gel electrophoresis of the Lp(a) and LDL purified from pooled plasma showed single bands in the Lp(a) and LDL position after staining with Fat Red 7B (supplementary Fig. 1A). The Lp(a) band was detected by both anti-apo(a) (supplementary Fig. 1B) and anti-apoB antibodies (supplementary Fig. 1C), while the LDL band was only detected by the anti-apoB antibody (supplementary Fig. 1C). Neither preparation showed contamination from other apoB-containing lipoproteins (supplementary Fig. 1C). A Western blot with an anti-apoA1 antibody (supplementary Fig. 1D) also showed no contamination with HDL.

### Lp(a) induces ABCA1 expression

**Fig. 1.**
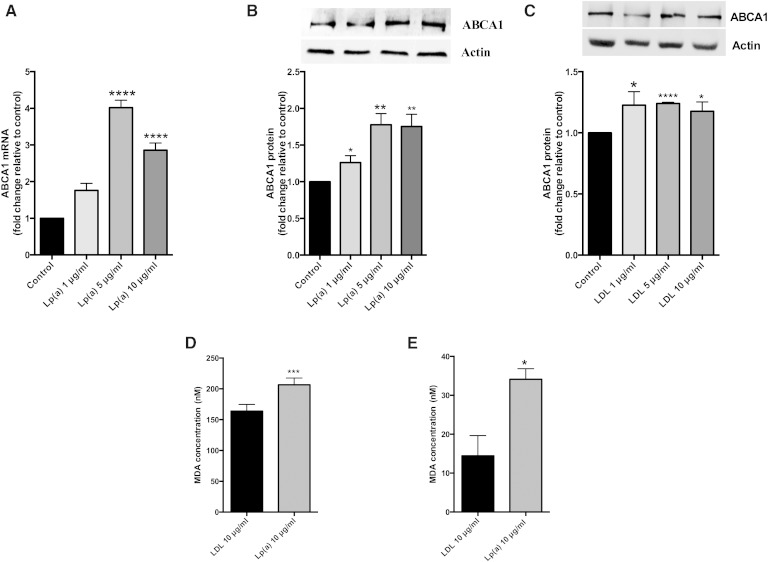
Lp(a) upregulates ABCA1 expression in HepG2 cells. HepG2 cells were treated with 1, 5, and 10 μg/ml purified Lp(a) or LDL protein for 12 h at 37°C. A: ABCA1 mRNA levels after treatment with Lp(a) as determined by RT-PCR. ABCA1 mRNA levels were normalized against β2-microglobulin and GAPDH mRNA and expressed relative to those of the control untreated cells. B: ABCA1 protein levels after treatment with Lp(a) as determined by Western blot. ABCA1 protein levels were normalized against actin (inset) and expressed relative to those of untreated cells. C: ABCA1 protein levels after treatment with LDL as determined by Western blot. ABCA1 protein levels were normalized against actin (inset) and expressed relative to those of untreated cells. D: MDA concentrations in 10 μg/ml Lp(a) protein and 10 μg/ml LDL protein as measured with the TBARS assay. E: MDA concentrations in 10 μg/ml Lp(a) protein and 10 μg/ml LDL protein after the addition of BHT/EDTA in the purification process as measured with the TBARS assay. Results are expressed as mean ± SE of at least two experiments performed in triplicate. *P < 0.05, **P < 0.01, ***P < 0.001, ****P < 0.0001 compared with control.

Treatment of HepG2 cells with 1 to 10 μg/ml of purified Lp(a) for 12 h resulted in a significant increase in ABCA1 mRNA levels up to 4.0-fold at 5 μg/ml and 2.8-fold at 10 μg/ml (**Fig. 1A**). ABCA1 protein levels were increased significantly by 1.3-fold at 1 μg/ml and 1.8-fold at 5 and 10 μg/ml Lp(a) (Fig. 1B). Treatment of HepG2 cells with LDL also stimulated ABCA1 expression, but not to the extent seen by Lp(a) and not in a concentration-dependent manner with ABCA1 protein levels, showing a 1.2-fold increase at 1, 5, and 10 μg/ml (Fig. 1C). We hypothesized that the greater response of ABCA1 to Lp(a) compared with LDL may be related to a higher oxidized lipid content. A TBARS assay to assess the MDA content of the purified Lp(a) and LDL samples showed the MDA content of Lp(a) to be significantly higher than that of LDL (206 nM versus 164 nM, respectively) (Fig. 1D). Addition of BHT and EDTA throughout the purification process gave a reduced MDA content in both lipoproteins albeit still higher in Lp(a) (34 nM versus 14 nM, respectively) (Fig. 1E). This indicates that MDA is mainly a measure of oxidized lipids generated in the purification process rather than innate content.

### Lp(a)-induced ABCA1 expression is under LXR regulation and stimulates cholesterol efflux

**Fig. 2.**
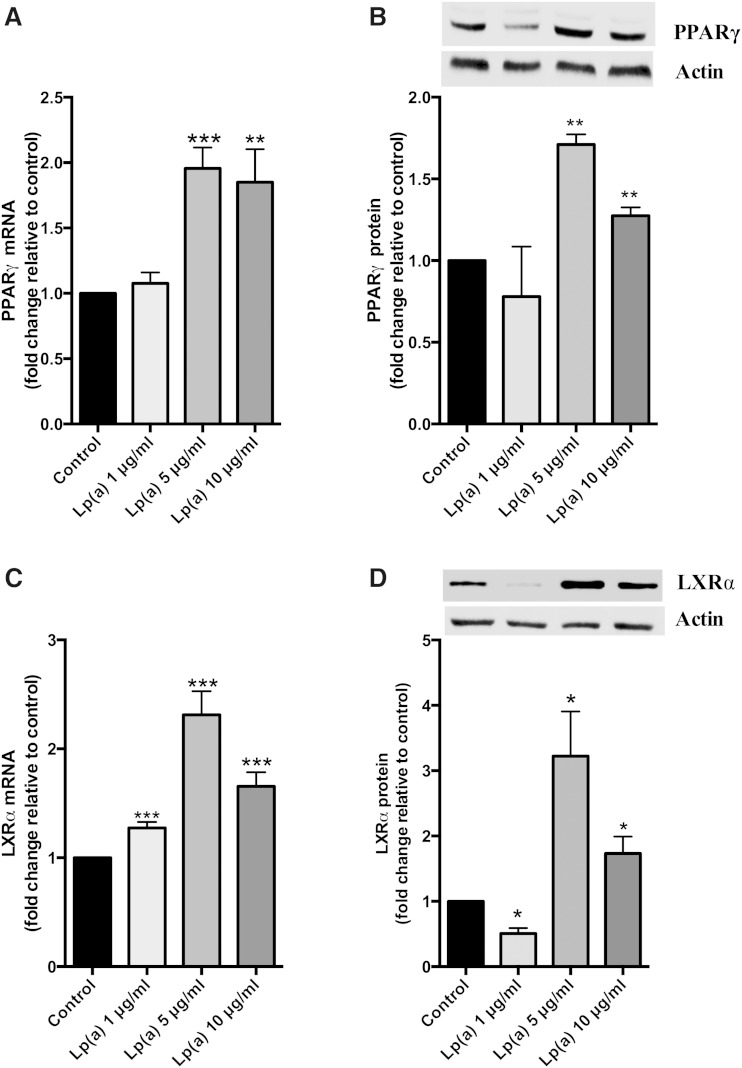
Lp(a) stimulates PPARγ-LXRα expression. HepG2 cells were treated with 1, 5, and 10 μg/ml purified Lp(a) protein for 12 h at 37°C. A: PPARγ mRNA levels as determined by RT-PCR. PPARγ mRNA was normalized to β2-microglobulin and GAPDH mRNA levels and expressed relative to those of the control untreated cells. B: PPARγ protein levels as determined by Western blot. PPARγ protein levels were normalized against actin (inset) and expressed relative to those of untreated cells. C: LXRα mRNA levels as determined by RT-PCR normalized to β2-microglobulin and GAPDH and expressed relative to control. D: LXRα protein levels normalized to actin and expressed relative to control. Results are expressed as mean ± SE for two experiments performed in triplicate for RT-PCR and triplicate Western blots for protein quantification. *P < 0.05, **P < 0.01, ***P < 0.001 compared with control.

To study the mechanism underlying the regulation of ABCA1 expression upon Lp(a) treatment, we measured the transcript levels of the PPARγ and LXRα transcription factors, which are known to upregulate ABCA1. The PPARγ transcript showed a significant increase up to 2-fold at 5 and 10 μg/ml Lp(a) (**Fig. 2A**). PPARγ protein levels were increased to 1.7-fold at 5 μg/ml and 1.3-fold at 10 μg/ml Lp(a) (Fig. 2B). The LXRα transcript was significantly elevated by 2.3-fold at 5 μg/ml and 1.7-fold at 10 μg/ml Lp(a) (Fig. 2C). This was associated with a significant increase in LXRα protein levels up to 3-fold at 5 μg/ml and 1.7-fold at 10 μg/ml (Fig. 2D). To further confirm the response of liver cells to Lp(a), we repeated the experiments shown in Figs. 1A, B and 2A–D in another hepatoma cell line, Hep3B. Incubation of Hep3B cells with 5 μg/ml Lp(a) also increased the ABCA1, PPARγ, and LXRα transcripts and proteins showing a similar (albeit lesser fold) response to that seen in the HepG2 cell line (supplementary Fig. 2).

**Fig. 3.**
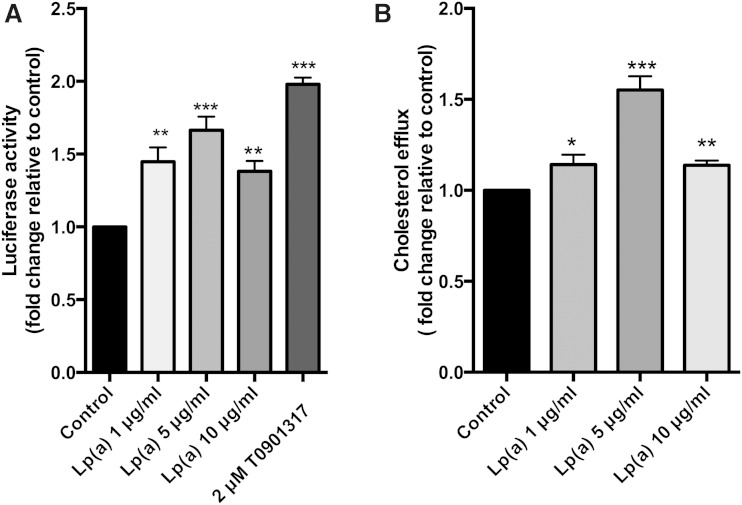
Lp(a) stimulates ABCA1 promoter activity and cholesterol efflux. A: ABCA1 promoter activity. HepG2 cells were transiently transfected with an ABCA1 promoter construct and promoter activity assessed by luciferase reporter assay after treatment with 1, 5, and 10 μg/ml Lp(a) protein. Luciferase fluorescence was normalized against the fluorescence from a Renilla transfection control and expressed relative to control untreated cells. B: Cholesterol efflux assays. HepG2 cells were loaded with [^3^H]cholesterol for 48 h prior to treatment with 1, 5, and 10 μg/ml Lp(a) protein. Cells were incubated with apoA1 acceptor for 2 h and apoA1-mediated cholesterol efflux calculated. Results are expressed as mean ± SE for at least two experiments performed in triplicate. *P < 0.05, **P < 0.01, ***P < 0.001 compared with control.

A luciferase promoter assay was performed to see whether the ABCA1 promoter was activated by Lp(a). The LXR agonist, T0901317 (2 μM), was included as a positive control. ABCA1 promoter activity was significantly increased up to 1.7-fold at 5 μg/ml Lp(a) and 1.4-fold at 10 μg/ml Lp(a) (**Fig. 3A**). To study whether the upregulation in the ABCA1 pathway by Lp(a) had any functional significance, we performed cholesterol efflux assays on the treated cells. Lp(a) treatment at 5 μg/ml promoted a 1.5-fold increase in cholesterol efflux onto apoA1 as compared with untreated cells. An increase in efflux was also mediated by 1 and 10 μg/ml Lp(a), but the increase was less than that seen at 5 μg/ml (Fig. 3B).

### Internalization of Lp(a) by HepG2 cells

**Fig. 4.**
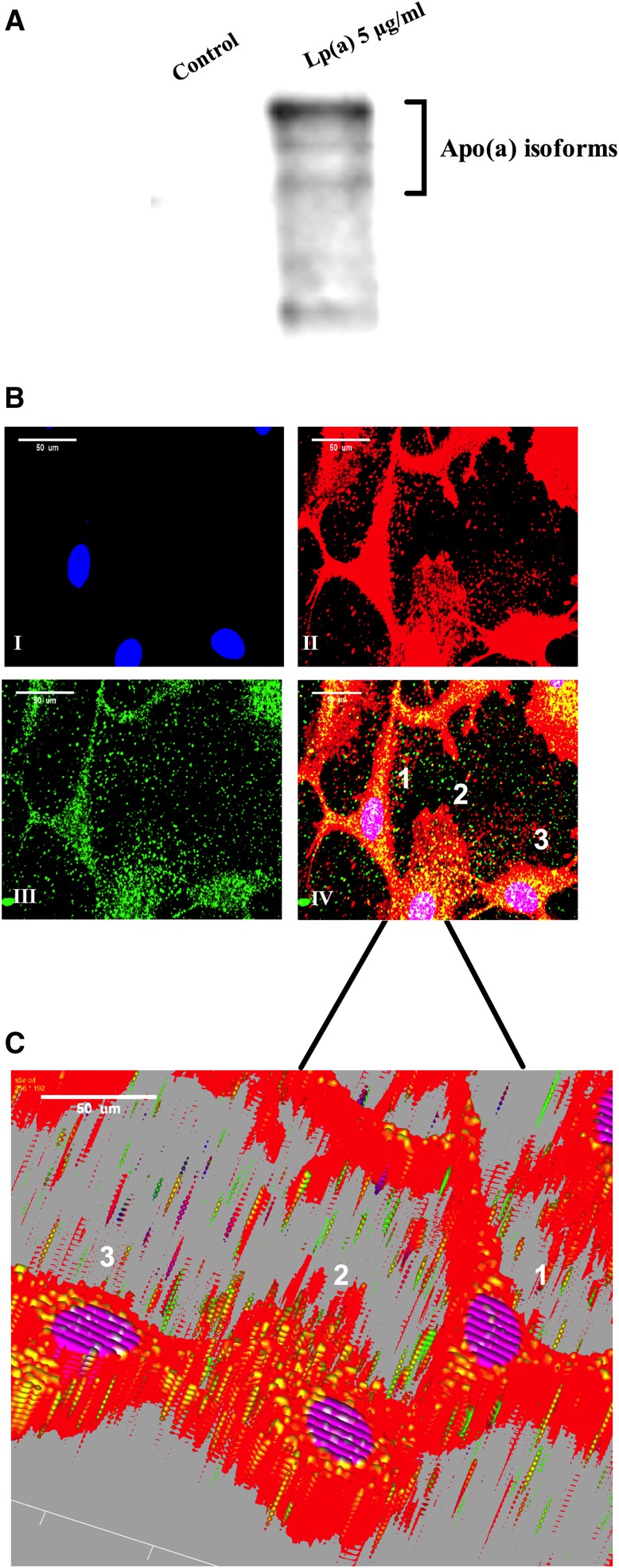
Lp(a) is internalized by HepG2 cells. A: Western blot of untreated control and 5 μg/ml Lp(a) protein-treated HepG2 cell lysates using an anti-apo(a) antibody. B: Confocal microscopy of Lp(a)-treated HepG2 cells stained with DAPI nuclear stain (I), AlexaFluor 594 WGA membrane stain (II), and the anti-apo(a) monoclonal antibody, a5, detected with AlexaFluor 488 anti-IgG (III). The merged image of all three stains is shown in (IV). Scale bar = 50 μm. C: 3D image after z stacking of the merged image. Ten slices of 0.5 μm thickness were taken through each cell and z-stacked using Olympus FluoView™ FV1000 Image Examiner software. In each case at least 100 cells were analyzed per condition across three independent experiments.

To check whether Lp(a) was being internalized, cell lysates from treated cells were subjected to Western blots with an anti-apo(a) monoclonal antibody (**Fig. 4A**). This showed the presence of multiple apo(a) bands (resulting from the multiple apo(a) isoforms in the pooled plasma used for Lp(a) isolation) indicative of Lp(a) uptake by cells. To confirm uptake further, confocal microscopy of treated cells was performed to visualize apo(a) within the cells (Fig. 4B). The apo(a) signal (green) was concentrated within the bounds of the cells stained by the WGA membrane-specific stain (red), giving rise to large areas of colocalization (indicated as yellow in the merged image). Z stacking of the merged confocal image was performed to confirm that apo(a) was found throughout the cell and not just at the cell surface (Fig. 4C). This showed the apo(a) signal (green) colocalized with the WGA signal (red) throughout the volume of the cell, confirming Lp(a) internalization. As the cells were permeabilized to allow internalization of the anti-apo(a) antibody, the WGA stain was also internalized leading to staining of membranous structures throughout the cell.

### SR-B1 mediates the ABCA1 response to Lp(a) through oxPLs

**Fig. 5.**
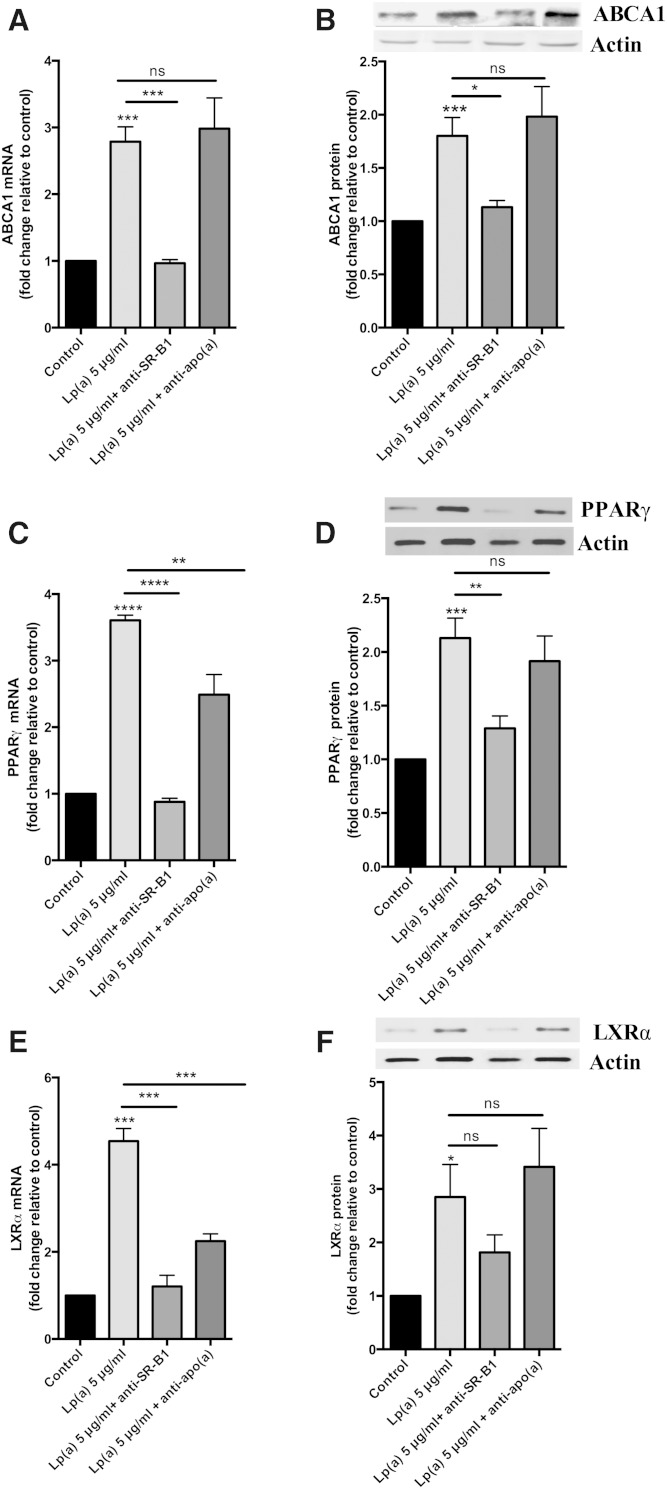
SR-B1 is involved in ABCA1 upregulation. HepG2 cells treated with 5 μg/ml Lp(a) protein were preincubated with an anti-SR-B1 antibody or the Lp(a) protein used for treatment preincubated with an anti-apo(a) polyclonal antibody and the effect on ABCA1, LXRα, and PPARγ mRNA transcript and protein levels determined. Transcript levels were normalized to β2-microglobulin and GAPDH and protein levels normalized to actin with all results expressed relative to the untreated control ABCA1 mRNA levels (A), ABCA1 protein levels (B), PPARγ mRNA levels (C), PPARγ protein levels (D), LXRα mRNA levels (E), and LXRα protein levels (F). Results are expressed as mean ± SE for two experiments performed in triplicate for RT-PCR and triplicate Western blots for protein quantification. *P < 0.05, **P < 0.01, ***P < 0.001, ****P < 0.0001 compared with control.

**Fig. 6.**
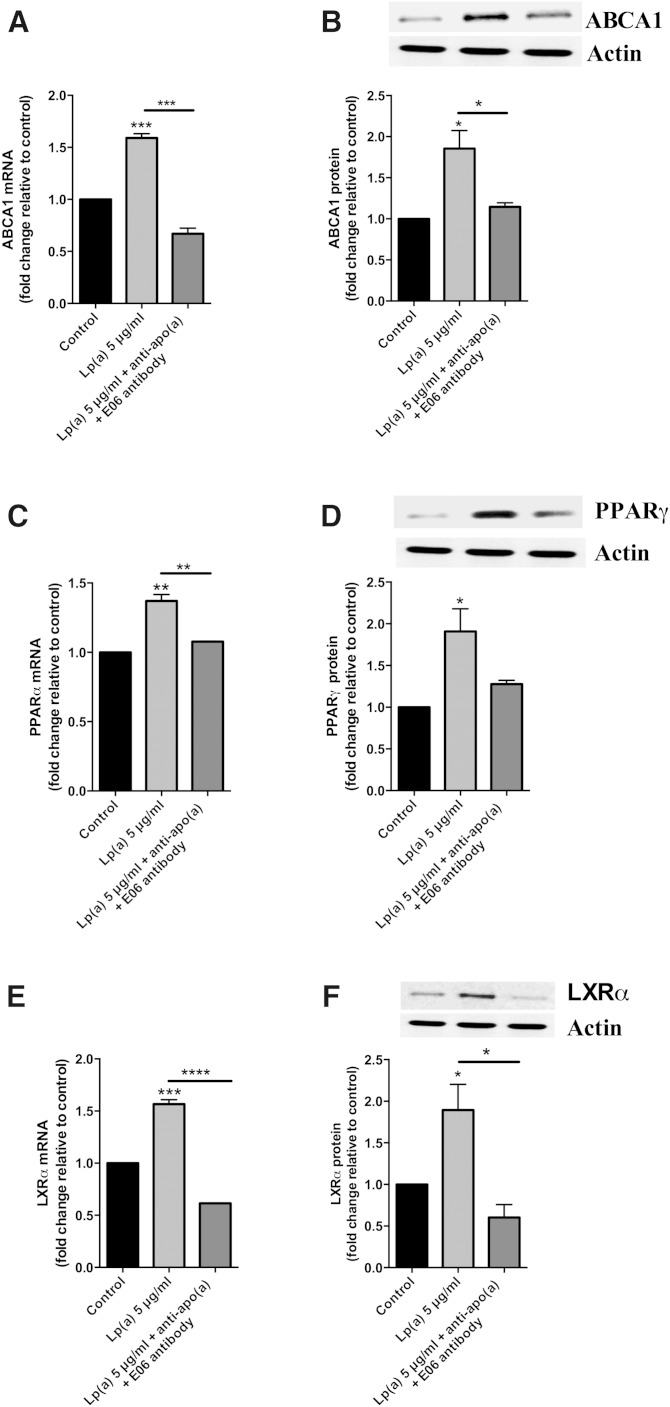
oxPLs of Lp(a) are involved in ABCA1 upregulation. HepG2 cells treated with 5 μg/ml Lp(a) protein were preincubated with an anti-apo(a) polyclonal antibody along with the E06 antibody and the effect on ABCA1, LXRα, and PPARγ mRNA transcript and protein levels were determined. Transcript levels were normalized to β2-microglobulin and GAPDH and protein levels normalized to actin with all results expressed relative to the untreated control ABCA1 mRNA levels (A), ABCA1 protein levels (B), PPARγ mRNA levels (C), PPARγ protein levels (D), LXRα mRNA levels (E), and LXRα protein levels (F). Results are expressed as mean ± SE of triplicates for RT-PCR and triplicate Western blots for protein quantification. *P < 0.05, **P < 0.01, ***P < 0.001, ****P < 0.0001 compared with control.

It has recently been reported that SR-B1 is a receptor for Lp(a) (22). Therefore, we investigated whether the ABCA1 response to Lp(a) was mediated by the SR-B1 receptor by using an antibody blocking approach. Preincubation of cells with an anti-SR-B1 antibody before Lp(a) treatment significantly reduced the ABCA1 response to Lp(a) treatment both at the transcript and protein level (**Fig. 5A, B**). Furthermore, the upregulation of the PPARγ and LXRα transcripts and proteins was significantly reduced in response to the anti-SR-B1 antibody (Fig. 5C–F). In contrast, preincubation with an anti-apo(a) to block whole Lp(a) particle uptake (assuming that this did not also effect the SR-B1-mediated uptake) had no effect on ABCA1 transcript and protein levels, with levels remaining similar to those observed in Lp(a)-treated HepG2 cells without antibody (Fig. 5A, B). The levels of the PPARγ and LXRα transcripts were reduced with anti-apo(a) treatment (Fig. 5C, E) but not to the extent seen with anti-SR-B1 treatment, and the protein levels of both PPARγ and LXRα were unaffected by the anti-apo(a) antibody treatment (Fig. 5D, F). Interestingly, a combination of anti-apo(a) and the E06 antibody which binds the oxPLs found in Lp(a) particles resulted in a significant decrease in the levels of ABCA1 transcript and protein, PPARγ transcript, and LXRα transcript and protein levels (**Fig. 6**). Indeed the decreases were similar to those seen with blocking SR-B1 (Fig. 5).

### SR-B1 mediates Lp(a) lipid, but not protein, uptake

**Fig. 7.**
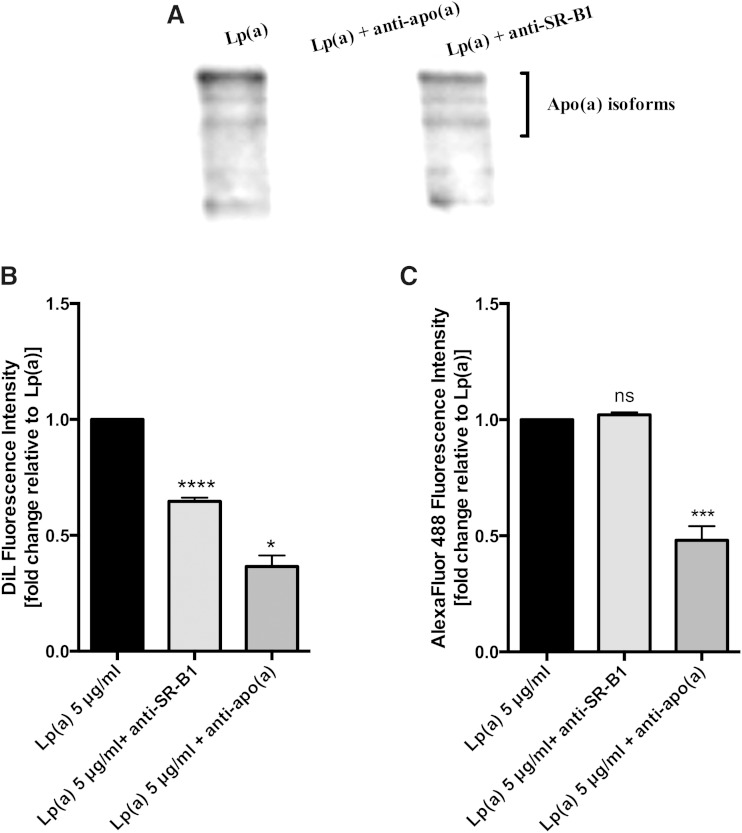
Inhibition of SR-B1 blocks Lp(a) lipid but not protein uptake. A: Western blot of Lp(a)-treated HepG2 cell lysates following preincubation of the cells with anti-SR-B1 or preincubation of the Lp(a) with an anti-apo(a) polyclonal antibody. The Western blot was performed with the anti-apo(a) monoclonal antibody, a5. B: Lipid uptake in 5 μg/ml Dil-labeled Lp(a) protein-treated cells following preincubation with an anti-SR-B1 antibody or preincubation of the Lp(a) with an anti-apo(a) antibody. The relative fluorescence intensity of Dil in cell lysates was measured at 549 nm excitation and 565 nm emission spectra. C: The apo(a) uptake in 5 μg/ml Lp(a) protein-treated cells preincubated with an anti-SR-B1 or the Lp(a) preincubated with an anti-apo(a) antibody was assessed. After treatment, cell lysates were incubated with an anti-apo(a) antibody and detected with AlexaFluor 488 IgG. The relative fluorescence intensity of AlexaFluor 488 at excitation spectra 500 nm and emission spectra at 520 nm was measured. Results are expressed as mean ± SE for at least two experiments done in triplicate. *P < 0.05, ***P < 0.001, ****P < 0.0001 compared with control.

To confirm whether both the anti-SR-B1 and anti-apo(a) antibodies had indeed blocked Lp(a) uptake, we visualized Lp(a) uptake by Western blot of cell lysates with an anti-apo(a) antibody (**Fig. 7A**). The SR-B1 antibody did not block the uptake of Lp(a), whereas the anti-apo(a) antibody completely blocked Lp(a) uptake. These results suggest that the ABCA1 response to Lp(a) might be mediated by selective lipid uptake from Lp(a) via SR-B1 and not whole particle uptake. To investigate this further, we labeled Lp(a) with Dil to label surface lipids and an anti-apo(a) antibody in combination with AlexaFluor 488 to label the protein component and then determined the effect of the SR-B1 antibody on lipid and protein uptake. The relative fluorescence intensity of Dil in cell lysates was reduced by 35% with anti-SR-B1 blocking (Fig. 7B), but the relative fluorescence of the protein component of Lp(a) showed no change in fluorescence intensity (Fig. 7C) indicating that the SR-B1 antibody was only affecting lipid transfer and not protein uptake. Treatment with the anti-apo(a) antibody, on the other hand, did block both lipid and protein uptake from Lp(a) into HepG2 cells, as indicated by a significant reduction in Dil and AlexaFluor 488 relative fluorescence (Fig. 7B, C) which we interpreted as a blocking of whole particle uptake.

To further confirm the role of SR-B1 in mediating the ABCA1 response to Lp(a), we also treated cells with the SR-B1 inhibitor, BLT-1. BLT-1 also significantly reduced the ABCA1 protein response (supplementary Fig. 3A). Furthermore, the relative fluorescence intensity of Dil in cells pretreated with BLT-1 before incubation with Dil-labeled Lp(a) was reduced by 35% (supplementary Fig. 3B). In contrast, the relative fluorescence of Lp(a) protein in cells, detected with an anti-apo(a) antibody and AlexaFluor 488, showed no change in fluorescence intensity with BLT-1 treatment (supplementary Fig. 3C). The results obtained with BLT-1 reiterated the results obtained with the SR-B1 antibody, showing that SR-B1 was mediating selective lipid transfer from Lp(a) and not protein uptake.

## DISCUSSION

Our study has uncovered a new connection between Lp(a) and HDL by demonstrating that Lp(a) upregulates the HDL production pathway in liver cells. We show that this effect is due to transfer of oxPLs from Lp(a) via the SR-B1 receptor leading to an upregulation of the PPARγ-LXRα-ABCA1 pathway and resulting in an enhanced cholesterol efflux to apoA1. This effect is specific to SR-B1 and independent of Lp(a) holoparticle uptake by liver cells.

Lp(a) has proven to be an important risk factor for developing CVD. Most trials report Lp(a) as having an effect on CVD independent of other plasma lipoproteins (2). Kinetics studies show that Lp(a) is metabolized differently to other apoB-containing lipoproteins (30), and proteomics studies report Lp(a) to have a unique protein composition (31). There are obvious connections between Lp(a) and LDL, as both molecules share similar functional properties with respect to their binding to structural proteins (32) and receptors from the LDLR family (33, 34). A connection between Lp(a) and HDL is less obvious, but there is some evidence that Lp(a) levels show a positive association with HDL in some populations (12–14). Here we aimed to investigate whether there was a direct connection between Lp(a) on HDL by testing the effect of Lp(a) on the ABCA1-mediated pathway of HDL production.

HepG2 cells were chosen for this study because the expression of ABCA1 in the liver is the major contributor to HDL production (8). Lp(a) was purified from a pooled plasma sample to avoid bias from working with just one or two apo(a) isoforms. We used LDL isolated from the same pooled sample as a control for these studies as it had been previously reported that LDL increases ABCA1 activity in macrophages (16). Incubation of HepG2 cells with Lp(a) showed a significant upregulation in ABCA1 at both the transcript and protein level, reaching a saturable effect by 5 μg/ml (Fig. 1A, B). In comparison, incubation of HepG2 cells with LDL showed the ABCA1 protein to be only modestly increased with no effect of concentration (Fig. 1C).

As Lp(a) showed a more robust response in upregulating ABCA1 compared with LDL, we considered that this might be due to a higher oxidized lipid content of Lp(a). ABCA1 expression is regulated by oxidized lipids (5) and Lp(a) is known to have a higher oxidized lipid content than LDL (35). Lp(a) is known to bind oxPLs via its apo(a) moiety (4). The catabolism of oxPLs is mediated by lipoprotein-associated phospholipase A2 which is enriched in Lp(a) compared with LDL (35). Lipoprotein-associated phospholipase A2 likely mediates the cleavage of oxPLs from Lp(a) to liberate oxidized free fatty acids. Oxidized lipids (both oxysterols and oxidized fatty acids) are known to upregulate the expression of ABCA1 via LXRα, in the case of oxysterols, and via PPAR-induced activation of LXRα, in the case of oxidized fatty acids (5). Investigation of the response of both transcription factors to Lp(a) showed that both were upregulated at the transcript and protein level in a concentration-dependent manner with the maximum protein response (1.7-fold for PPARγ and 3-fold for LXRα) reaching saturation at 5 μg/ml (Fig. 2A–D). Furthermore, this response was associated with a 1.7-fold increase in ABCA1 promoter activity (Fig. 3A). These results suggest that the LXRα-mediated upregulation of ABCA1 was responsible for the Lp(a) response. This is in contrast with the previously reported ABCA1 response to LDL in macro­phages, which was shown to be mediated by SP1 not LXRα (16).

The ABCA1 response elicited by Lp(a) was associated with a significantly increased cholesterol efflux (Fig. 3B) indicative of a functional effect. Whether the Lp(a)-mediated upregulation of ABCA1 and associated increase in cholesterol efflux is of physiological significance with respect to increasing HDL levels is unknown. It is difficult to translate the Lp(a) concentration which promoted the response in isolated liver cells, i.e., 5 μg/ml with the equivalent concentration required to promote a response in vivo. Our results, however, would suggest an Lp(a) concentration at the low end of the physiological range [Lp(a) levels range from 0 to 200 mg/dl with 80% of subjects having a level below 50 mg/dl] (2). Our study would also suggest that the effect of Lp(a) on ABCA1 is saturable and might fail to be further promoted by higher concentrations of Lp(a). It is tempting to speculate that a positive effect of Lp(a) at low levels on HDL production from the liver might contribute to the bottom of the J-shaped curve seen between Lp(a) level and CVD risk (35) where low levels do not increase risk.

We hypothesized that a receptor-mediated uptake of Lp(a) would be necessary to elicit the effect on ABCA1. Western blots of lysates from Lp(a)-treated cells showed the presence of multiple apo(a)-sized isoforms suggesting uptake of all isoforms present (Fig. 4A). Immunohistochemistry studies showed Lp(a) to be present throughout the liver cells colocalizing with membranous fractions throughout the cells, as shown by Z-stacking (Fig. 4C). Various receptors have been reported to bind Lp(a), including LDLR (33), LDL receptor-related protein (LRP) (34), megalin (36), VLDLR (20), as well as asialoglycoprotein receptors (ASGPR) (21), plasminogen receptors (37), and more recently SR-B1 (22). Neither VLDLR nor megalin are expressed by the liver (20, 36), and evidence suggests Lp(a) is a reasonably poor ligand for LDLR and LRP (34). As the LXR-ABCA1 pathway is activated by oxidized lipids which are a well-known ligand for the SR-B1 receptor (38), and Lp(a) is known to specifically accumulate oxPLs (35), we therefore investigated the role of SR-B1 in eliciting the Lp(a)-mediated ABCA1 response. Indeed, antibody-mediated blocking of the SR-B1 receptor effectively blocked the PPAR-LXR-ABCA1 response to Lp(a) (Fig. 5). Interestingly, labeling of the lipid and protein component of Lp(a) showed that blocking SR-B1 (either by the SR-B1 antibody or by the SR-B1 inhibitor, BLT-1) only affected the uptake of Lp(a) lipid, not protein, suggesting that SR-B1 mediates selective lipid uptake from Lp(a). Indeed Yang et al. (22) have recently shown that SR-B1 does mediate selective uptake of lipids from Lp(a). Further support for this was gained when an experiment using the E06 antibody, which binds to oxPLs, showed effective blocking of the ABCA1 response (Fig. 6A, B). Collectively our results show that the selective uptake of oxPLs from Lp(a) is the effector of the ABCA1 response.

Although we have identified selective lipid uptake from Lp(a) via SR-B1 as being responsible for the ABCA1 response, our results clearly showed there was an independent uptake of apo(a) into liver cells. This was effectively blocked by an anti-apo(a) antibody which blocked both the Lp(a) lipid and apo(a) protein component indicative of whole Lp(a) particle uptake. The receptor(s) responsible for whole Lp(a) particle uptake in the liver are not certain. The LDLR, LRP, asialoglycoprotein, and plasminogen receptors are all potential candidates. Both LDLR and LRP bind to apoB and apoE moieties on the LDL particle (39), although these may be masked by the presence of apo(a) in Lp(a). The fact that an apo(a) antibody blocked the whole Lp(a) particle uptake may hint at the plasminogen receptor, as this receptor specifically interacts with the apo(a) component of Lp(a) (37). Alternatively, the asialoglycoprotein receptor which interacts with N-glycosylated residues on apo(a) (21) could also be involved.

Whether it is solely the lipid component of Lp(a) that interacts with SR-B1 for uptake or whether apo(a) or apoB is involved is not known. Interestingly, blocking with an apo(a) antibody did not affect the selective lipid uptake from Lp(a) (Fig. 7C) suggesting that apo(a) may not be involved.

From a clinical perspective, we expect that the ABCA1 response mediated by Lp(a) might show variation between individuals depending on the lipid and protein composition of individual Lp(a) particles. The oxPL content of Lp(a) particles varies (40), as does the protein make-up as recently shown by a quantitative proteomic study of individual Lp(a) particles (41). Indeed, the response could vary within an individual because most individuals have two apo(a) isoforms giving rise to two different species of Lp(a) particle which may differ in protein and lipid composition. Although our results show that Lp(a) upregulates HDL production, these results still need to be proven in vivo. Another alterative hypothesis to consider in vivo is that Lp(a) may compete with HDL for binding to SR-B1, thus reducing the selective uptake of HDL lipids and increasing HDL levels.

In conclusion, we have uncovered a novel connection between Lp(a) and HDL showing that Lp(a) upregulates ABCA1 in liver cells via selective uptake of oxPLs from Lp(a) by SR-B1. Uncovering this response provides new insight into Lp(a) metabolism by liver cells and may prompt further investigation into the associations between Lp(a) and HDL in clinical settings.

## Supplementary Material

Supplemental Data
