# Supplementary material for: Lipoprotein (a) upregulates ABCA1 in liver cells via scavenger receptor-B1 through its oxidized phospholipids
Source: J Lipid Res. 2015 Jul;56(7):1318–28. doi: 10.1194/jlr.M056150 (PMC4479336; doi:10.1194/jlr.M056150)
Supplement: Supplemental Data [file supp_56_7_1318__index.html]

Lipoprotein(a) upregulates ABCA1 in liver cells via scavenger receptor-B1 through its oxidised phospholipids. — Lipoprotein (a) upregulates ABCA1 in liver cells via scavenger receptor-B1 through its oxidized phospholipids — Supplemental Data 

# Lipoprotein (a) upregulates ABCA1 in liver cells via scavenger receptor-B1 through its oxidized phospholipids

## Supplemental Data

**Files in this Data Supplement:**

- Supplementary Data - Supplementary data including figures and table
