# Supplementary material for: Lipoprotein (a) upregulates ABCA1 in liver cells via scavenger receptor-B1 through its oxidized phospholipids
Source: J Lipid Res. 2015 Jul;56(7):1318–28. doi: 10.1194/jlr.M056150 (PMC4479336; doi:10.1194/jlr.M056150)

Supplementary Table 1: Sequences of gene specific primers used for RT-PCR.

Gene	Forward Primer (5'-3')	Reverse Primer (5'-3')
ABCA1	GGTAGGAGAAAGAGACGCAAAC	AACAAAACAATAACGCCCAAGT
β-2M	TTTCATCCATCCGACATTGA	CCTCCATGATGCTGCTTACA
LXRα	CGGGCTTCCACTACAATGTT	TCAGGCGGATCTGTTCTTCT
PPARγ	GTGTTGGTGATAGGTCCGAAAT	CCCAAGTGAATTGGATTCTTCT
GAPDH	GCACCCTGGTCTGAGGTTAAAT	AGGAGTGGGAGCACAGGTAAG

Supplementary figures:

Supplementary Figure 1: Agarose gel electrophoresis of purified Lp(a) and LDL. (A) Gel stained with Fat Red 7B. (B) Western blot of gel with anti-apo(a) antibody. (C) Western blot of gel with anti-apoB antibody. (D) Western blot of gel with anti-apoA1.

Supplementary Figure 2: Lp(a) upregulates ABCA1, PPARγ and LXRα in Hep3B cells. Hep3B cells were treated with 5 μg/ml purified Lp(a) protein for 12 hours at 37° C. (A) ABCA1 mRNA levels after treatment with Lp(a) were determined by RT-PCR. mRNA levels were normalized against β2-microglobulin and GAPDH mRNA and expressed relative to that of control untreated cells. (B) ABCA1 protein levels were determined by western blot. Protein levels were normalized against actin and were expressed relative to control. (C) PPARγ mRNA levels, (D) PPARγ protein levels, (E) LXRα mRNA levels, (F) LXRα protein levels. Results are expressed as mean ± S.E of triplicates for RT-PCR and triplicate western blots for protein quantification. *, $p < 0.05$ **, $p < 0.01$ ***, $p < 0.001$ compared with control.

Supplementary Figure 3: SR-B1 inhibitor, BLT-1 blocks ABCA1 protein expression and lipid uptake from Lp(a): (A) HepG2 cells treated with 5 μg/ml Lp(a) were preincubated with 100 μM of BLT-1 and the effect on ABCA1 protein levels determined. ABCA1 protein levels were normalised to actin and expressed relative to the untreated control. (B) Lipid uptake in cells treated with 5 μg/ml Dil-labelled Lp(a) after preincubation with 100 μM of BLT-1. The relative fluorescence intensity of Dil in cell lysates was measured at 549 nm excitation and 565 nm emission spectra. (C) Apo(a) uptake in cells treated with 5 μg/ml Lp(a) after preincubation with 100 μM of BLT-1. After treatment, cells lysates were incubated with an anti-apo(a) antibody and detected with AlexaFluor 488 IgG antibody. The relative fluorescence intensity of AlexaFluor 488 at excitation spectra 500 nm and emission spectra at 520 nm was measured. Results are expressed as mean ± S.E of triplicates of at least one experiment. *, $p < 0.05$, ****, $p < 0.0001$ compared with control.

Supplementary Figure 1

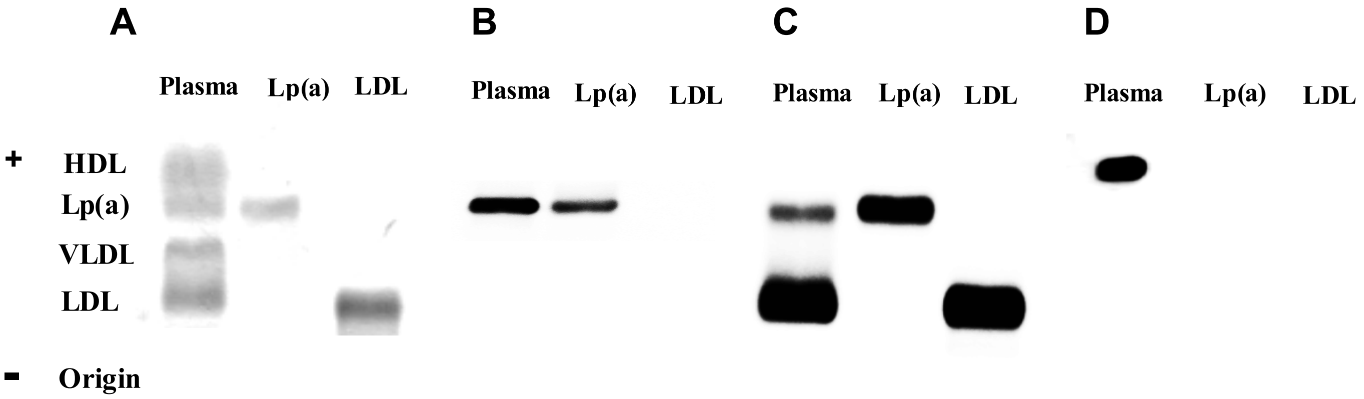

Supplementary Figure 2

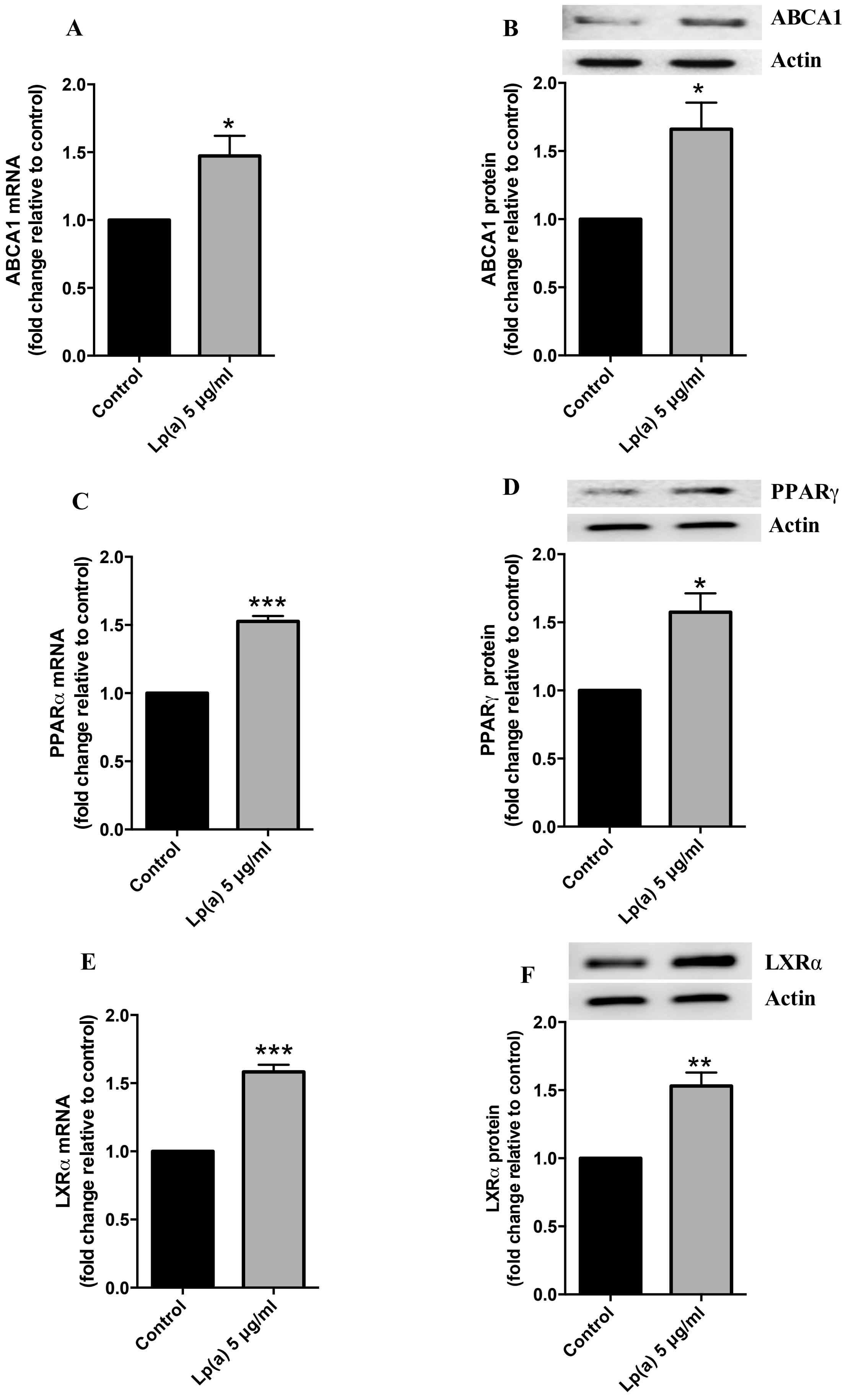

Supplementary Figure 3

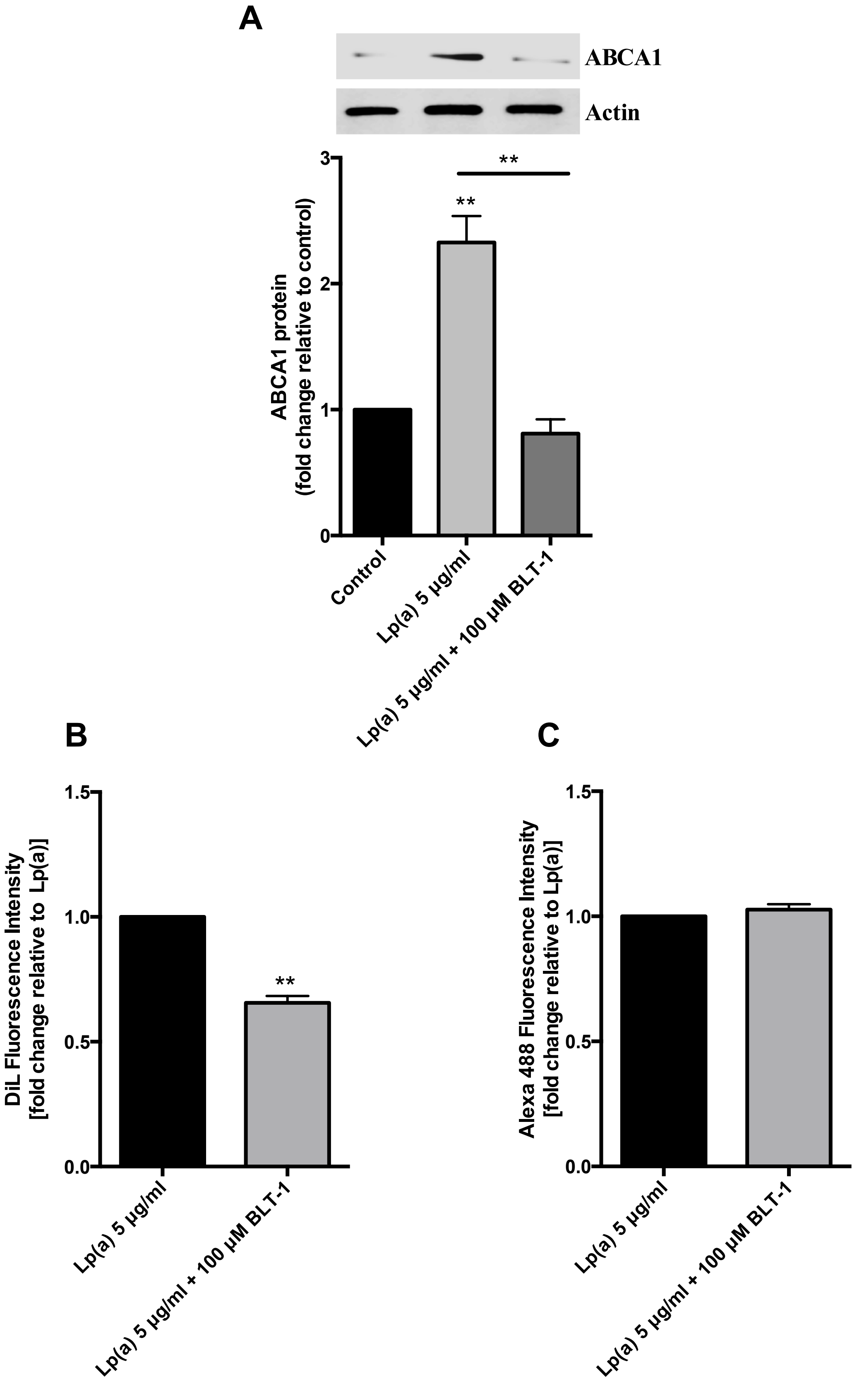

Supplement: Supplemental Data [file supp_M056150_jlr.M056150-1.pdf]
